# Mine or my neighbours’ offspring: an experimental study on parental discrimination of offspring in a colonial seabird, the little auk *Alle* *alle*

**DOI:** 10.1038/s41598-023-41925-5

**Published:** 2023-09-12

**Authors:** Dorota Kidawa, Katarzyna Wojczulanis-Jakubas, Dariusz Jakubas, Rupert Palme, Mateusz Barcikowski

**Affiliations:** 1 Department of Vertebrate Ecology and Zoology, Faculty of Biology, University of Gdańsk, ul. Wita Stwosza 59, 80-308 Gdańsk, Poland; 2 Department for Biomedical Sciences, University of Veterinary Medicine, Veterinärplatz 1, 1210 Vienna, Austria

**Keywords:** Ecology, Evolution, Neuroscience, Physiology, Psychology, Zoology, Ecology

## Abstract

Individual recognition (IR) abilities may result from various ecological and naturally selected features of a species. Complex IR mechanisms should develop when the risk of misidentification of a chick is high. For colonial seabirds, the ability to identify their own brood is crucial to ensure parental fitness. Vocalizations seem to be a key component of most parent–offspring interactions, although few studies have assessed the interindividual differences in seabird chick calls and their potential usage in IR. The little auk (*Alle* *alle*), which breeds in dense colonies, constitutes a perfect model for testing IR. In this study, we (1) examined chick calls at different stages of the nesting period, and (2) cross-fostered chicks to examine the rate of acceptance/nonacceptance of chicks by parents. We found significant interindividual differences in chick begging and fledging calls. Surprisingly, all cross-fostered chicks in our experiments were accepted by their foster parents, and male parents were as equally likely to accept cross-fostered chicks as females, even though the sexes would be expected to differ in offspring recognition due to different postfledging interactions with the chick. The revealed individuality of chick calls suggests the potential for chick vocal recognition in the studied species, but parent birds may disregard the individual characteristics enabling chick discrimination. This may take place as long as the chick is found in the nest because of the high likelihood that the chick present there is the focal one. However, IR during and after fledging requires further study. Studying the complexity of IR mechanisms is important for better understanding various avian social relationships and interactions.

## Introduction

Parents’ ability to distinguish between their own and foreign offspring is a strong naturally selected function, as parental effort allocated to nonkin progeny naturally decreases the fitness of the parents^1–3^. Thus, adults are expected to focus their parental attention on their own offspring, ignoring any other unrelated individual, to ensure their own reproductive success^4,5^. In contrast to adults, chicks would benefit from any parental care; therefore, a chick’s discrimination by the parent ought to be more important than mutual recognition. This may be particularly relevant in species where there is a high risk of offspring misidentification and a high cost of a possible mistake. A good example is colonially nesting seabirds inhabiting severe polar regions. Breeding in colonies increases the risk of offspring misidentification, while parental care is extraordinarily costly (e.g.^6,7^). In addition, brood size is often restricted to a single chick or two chicks; thus, for parents, maximizing the chance of survival of their own offspring should be particularly important^8–12^.

It is possible that in some species, chicks can be identified by simple topographic cues of the nest. However, even in these species, individual recognition (IR) is likely to be present, especially when the chicks become more mobile and leave the nest for short periods, as well around and after fledging, when chicks still depend on parental care^8,13,14^. Mechanisms of individual recognition on the parent–offspring axis are diversified and not fully recognized. How animals recognize themselves depends on species-specific individual cues and behavioural patterns. One or more of the senses, such as smell, sight, or hearing, or a combination of them are usually employed in IR^15^. Chemical signals are often used by species that are nocturnal at their breeding sites, e.g., blue petrels (*Halobaena* *caerulea*), which use smell to return to the colony at night and locate the nest site and the offspring within^16^. Visual cues are useful in open landscapes, where physical barriers blocking signal transmission are not an issue. In many species, IR is acoustic signal-dependent^17^, which is quite efficient for individual identification regardless of the environmental circumstances.

Differentiated call structures, enabling chick discrimination, should be present in colonially breeding species where chicks do not remain in an easily distinctive nest^18^, and thus, the chance of chick intermingling is high. For instance, in Antarctic penguins (*Aptenodytes*, *Pygoscelis* or *Eudyptes*), in which all nests are open and near each other, parent–offspring vocal recognition is common and well known^9–12^. In thick-billed guillemots (*Uria* *lomvia*) breeding in high density on ledges, the parents are also able to recognize their chicks vocally^8^. Razorbills (*Alca* *torda*), close guillemot relatives, have more discrete and less dense breeding sites, but there is still a risk of chick intermingling, especially during fledging at approximately the 15th day of life^19^. Interestingly, male but not female parents recognize the call of 10-day-old chicks and provide postfledging parental care at sea^20^.

In this study, we investigated the question of offspring recognition in the little auk (or dovekie; *Alle* *alle*), which is a clearly colonial, semiprecocial seabird with long and extensive parental care over a single offspring. Both parents share incubation and chick rearing^21^. The offspring depart the colony at approximately the 24th–25th day of life, escorted by the male parent^22,23^, as in the razorbill^20^. These species characteristics create an excellent study system for parent–offspring communication and IR^24^. Little auks build distinct nests; thus, the selection on IR is possibly not that strong; the chick could be found based on the topographic cues of the nest. However, nest density is very high in most parts of the colony. Some nests are even connected with each other by the chambers underneath, and rare observations indicate that chicks may visit the neighbouring nests (*personal* *observation*). There is also some genetic evidence of little auk chicks not being related to both parents, perhaps due to switches between nests^25^. Later in the season, chicks are more mobile and may move among the nests, wandering around on the colony’s surface or underneath, especially when the parents neglect their feeding duties or when chicks attempt to hide from predators. For instance, chicks scared by glaucous gulls (*Larus* *hyperboreus*) may attempt to hide among rock debris^26^. Young birds during the prefledging period spend considerable time wandering around their nest, exercising their wings and waiting for their parents to come with food. Finally, parent–offspring recognition may be crucial at the fledging event, allowing the fledgling and the accompanying parent (male) to travel together and to reunite when they accidentally separate or as a consequence of predator attack. It may also be important later at sea, when the male parent takes care of the chick^21^.

Since the little auk is a very vociferous species, we first investigated whether bioacoustic parameters may code the individual identity of the chick (critical for IR). Then, in the cross-fostering experiment, we tested parental acceptance/nonacceptance of chicks switched between nests. We hypothesized the following:

1. Chicks differ in the acoustic parameters of their calls; due to ontogenetic processes, those differences are most evident at the end of the nesting period.
2. Due to the potentially significant interindividual differences in little auk chick calls and high cost of chick misidentification, parent birds do not accept a cross-fostered chick.
3. Due to their role in postfledging care at sea, little auk males are expected to have more pronounced chick IR than females, as in the razorbill, and be more discriminating against cross-fostered chicks.

## Results

### Chick vocalization

**Figure 1:**
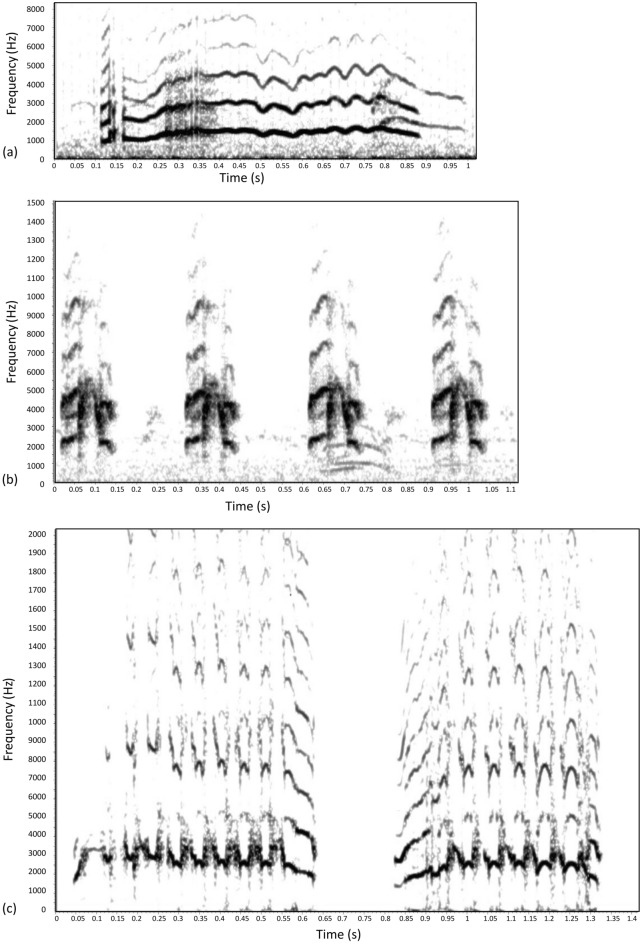
Examples of little auk chick calls. (**a**) Begging call in the 1st week of chick life. (**b**) Begging call in the 4th week of chick life. (**c**) Fledging call. Spectrograms were created in RAVEN Pro 1.6.1^51^.

The begging calls of chicks in their 1st week of life were characterized by a longer syllable duration and a lower frequency range than the begging calls of the older chicks (Table 1, Fig. 1a,b, Supplementary Table 1). Begging calls recorded in the 4th week of chick life were more “rhythmic” (shorter in duration but more frequent) and more chaotic in regards to energy distributions on the time and frequency spectra, as they had higher spectrographic entropy measures than those in the 1st week (Table 1, Fig. 1a,b, Supplementary Table 1). The intraclass correlation coefficient (ICC) of a random effect (chick) was relatively high (0.50–0.80) for all of the parameters (Supplementary Table 1). The fledging call was characterized by a long syllable duration and high frequency ranges (Table 1, Fig. 1c).

**Table 1:** Descriptive statistics of little auk chick begging calls in the 1st (n = 538) and 4th (n = 100) weeks of life and fledging calls (n = 446).

	Begging calls in 1st week^1^					Begging calls in 4th week^2^					Fledging calls^3^				
	Min	Q_1_	Median	Q_3_	Max	Min	Q_1_	Median	Q_3_	Max	Min	Q_1_	Median	Q_3_	Max
Duration (s)	0.07	0.13	0.26	0.49	0.92	0.04	0.10	0.11	0.12	0.14	0.18	0.30	0.33	0.40	0.80
F min (Hz)	340	625	730	879	1261	951	1096	1201	1300	1689	178	1124	1217	1342	2137
F Q_1_ (Hz)	1035	1369	1547	1752	2356	1708	1941	2076	2361	3501	1790	2339	2468	2591	3308
F medium (Hz)	1060	1523	1714	1974	2615	1967	2173	2334	2822	4020	1942	2561	2727	2933	4121
F Q_3_ (Hz)	1089	1597	1858	2092	2829	2063	2416	2588	3291	4539	2078	2811	3029	3276	4174
F max (Hz)	1470	1898	2129	2370	3229	2279	2745	3050	3966	5209	2245	3265	3563	3899	6729
F IQR (Hz)	31	144	239	411	819	231	421	597	820	1289	86	409	558	709	1901
F peak (Hz)	216	1513	1686	2032	2810	1773	2118	2291	3329	5231	1945	2464	2637	2897	5404
Entropy	0.61	0.74	0.79	0.83	0.92	0.73	0.83	0.85	0.87	0.91	0.67	0.79	0.80	0.82	0.87

The begging (both in the 1st and 4th weeks of life) and fledging calls were characterized by high individual repeatability for most of the acoustic parameters (Table 2). The individual repeatability of the maximum frequency measures was high in all analysed calls, while the minimum frequency measures had the lowest repeatability in all analysed calls (Table 2). Additionally, begging calls in the 1st and 4th weeks of life had a high (> 0.80) individual repeatability of call duration, entropy, and medium and Q_3_ frequency measures.

**Table 2:** Individual repeatability of the acoustic parameters of little auk chick begging calls in the 1st (n = 538) and 4th (n = 100) weeks of life and fledging calls (n = 446). The highest repeatability measures (R > 0.8) and significant (p < 0.05) effects are bolded.

Parameter	Begging calls in 1st week^1^			Begging calls in 4th week^2^			Fledging calls^3^		
	R	CI	*p*	R	CI	*p*	R	CI	*p*
Duration (s)	**0.90**	**0.74**–**0.95**	**< 0.001**	**0.88**	**0.70**–**0.94**	**< 0.001**	0.75	0.54–0.85	**< 0.001**
F min (Hz)	0.70	0.42–0.83	**< 0.001**	0.58	0.25–0.76	**< 0.001**	0.42	0.22–0.58	**< 0.001**
F Q_1_ (Hz)	0.77	0.51–0.88	**< 0.001**	0.64	0.32–0.80	**< 0.001**	0.64	0.41–0.77	**< 0.001**
F medium (Hz)	**0.83**	**0.60**–**0.91**	**< 0.001**	**0.89**	**0.72**–**0.95**	**< 0.001**	0.62	0.39–0.76	**< 0.001**
F Q_3_ (Hz)	**0.84**	**0.62**–**0.92**	**< 0.001**	**0.93**	**0.80**–**0.96**	**< 0.001**	0.66	0.44–0.79	**< 0.001**
F max (Hz)	**0.84**	**0.63**–**0.92**	**< 0.001**	**0.94**	**0.83**–**0.97**	**< 0.001**	**0.82**	**0.65**–**0.90**	**< 0.001**
F IQR (Hz)	0.73	0.45–0.85	**< 0.001**	0.73	0.44–0.86	**< 0.001**	0.56	0.33–0.71	**< 0.001**
F peak (Hz)	0.68	0.39–0.82	**< 0.001**	**0.89**	**0.71**–**0.94**	**< 0.001**	0.39	0.19–0.56	**< 0.001**
Entropy	**0.82**	**0.59**–**0. 91**	**< 0.001**	**0.81**	**0.57**–**0.91**	**< 0.001**	0.33	0.16–0.50	**< 0.001**

The structures of chick begging calls were significantly more similar (pairwise spectrogram cross-correlations and Mantel test) within the same individual than between different individuals in the 1st week of chick life (r = 0.31, p < 0.001, n = 538), in the 4th week of chick life (r = 0.53, p < 0.001, n = 100), and the same was true for chick fledging calls (r = 0.53, p < 0.001, n = 446).

The Beecher information statistic was the highest for the begging calls in the fourth week of chick life (H_s_ = 5.57 for all the variables). It was slightly lower for the begging calls in the first week of chick life (H_s_ = 4.81) and lowest for the fledging calls (H_s_ = 2.27). Consequently, a maximum of 28, 48 and 5 different individuals could be identified based on the study parameters during the first week, fourth week and fledging, respectively.

### Cross-fostering experiment

**Figure 2:**
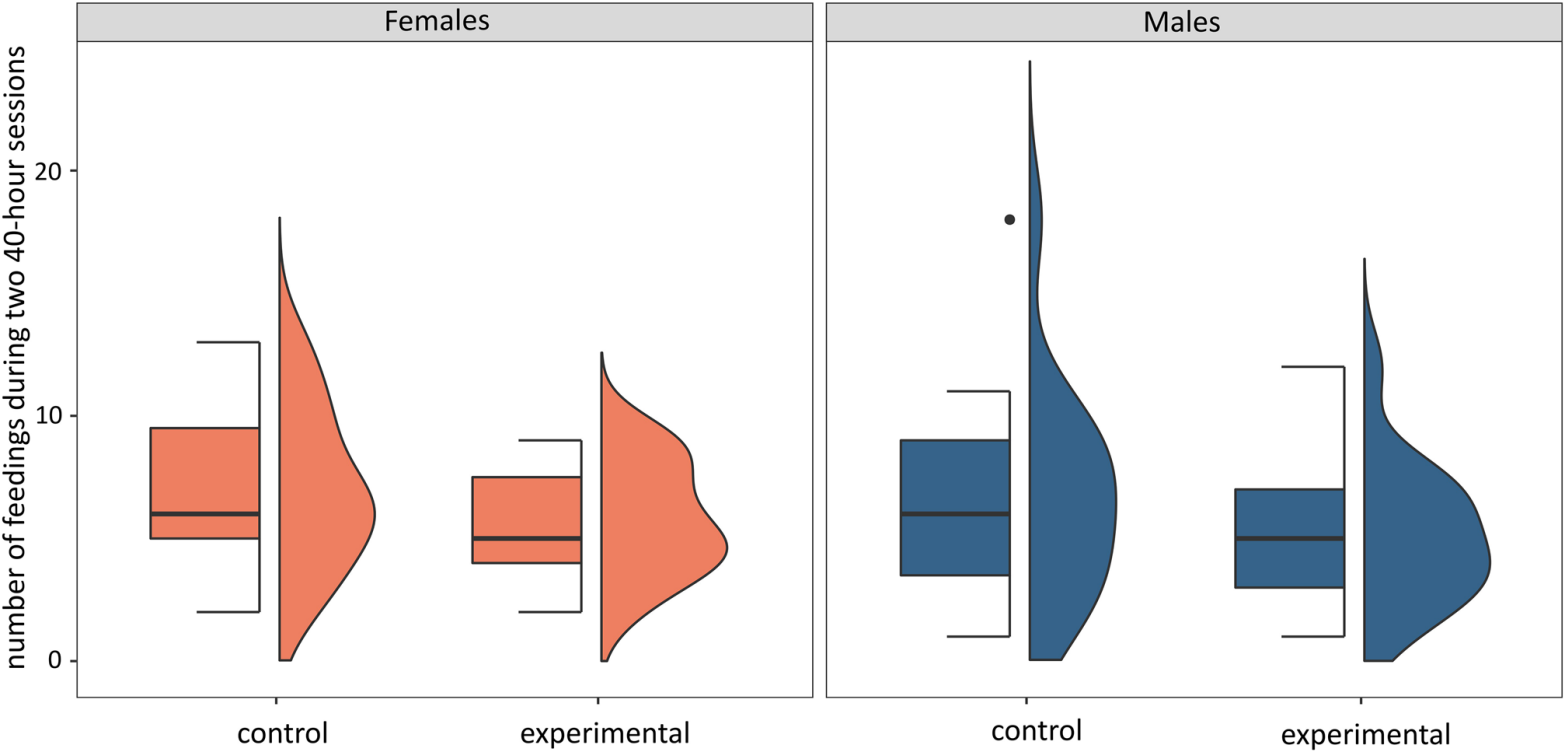
Number of feedings performed during two 40-h recording sessions: before the experiment (control) and after the cross-fostering experiment (experimental) in males and females. Boxplots show the median (band inside the box), the first (25%) and third (75%) quartiles (box), the lowest and the highest values within 1.5 interquartile range (whiskers), and outliers (dots). Right side density plots show the distribution of data.

**Figure 3:**
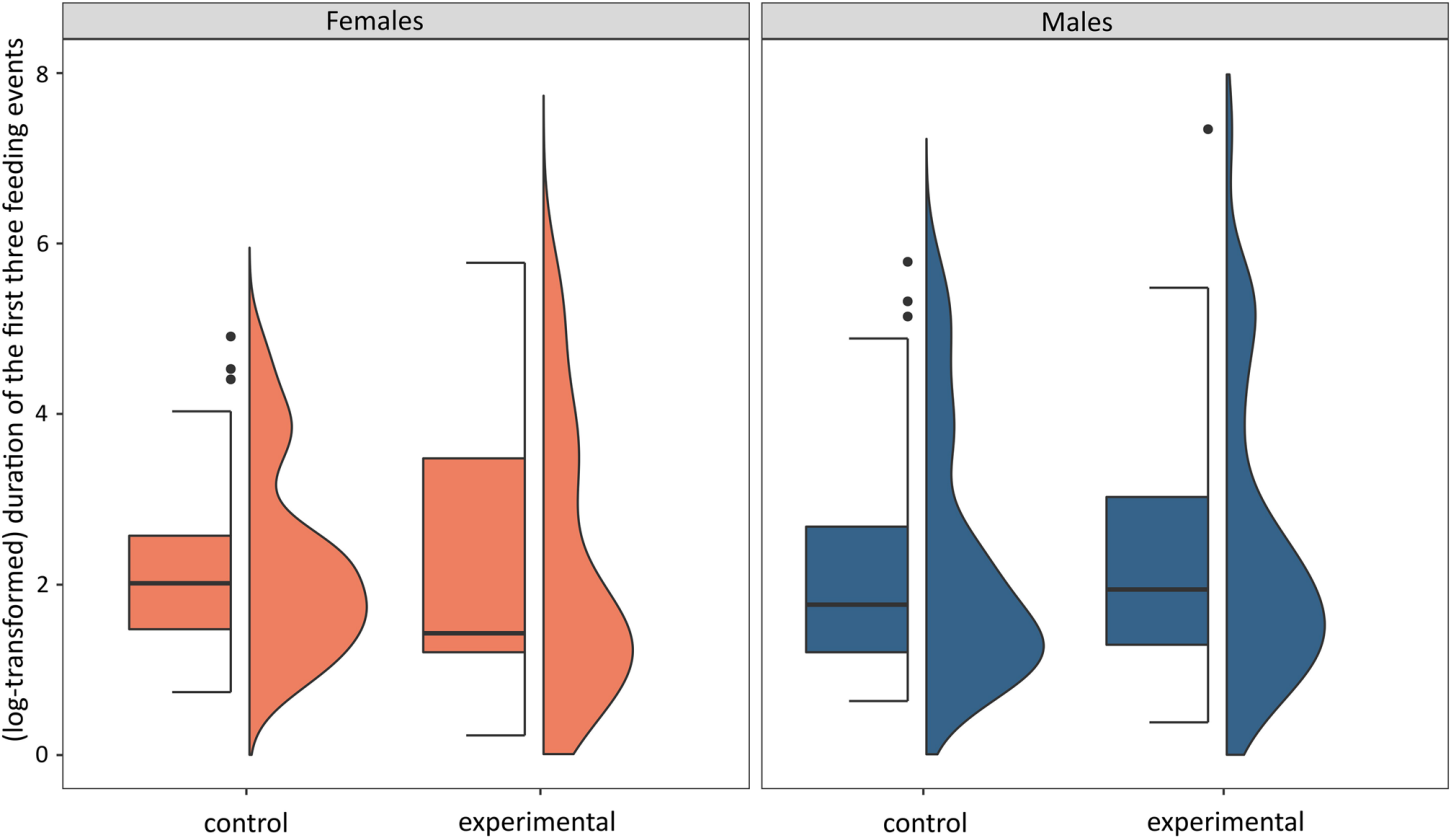
Duration of the first three feeding events (log-transformed): before the experiment (control) and after the cross-fostering experiment (experimental) in males and females. Boxplots show the median (band inside the box), the first (25%) and third (75%) quartiles (box), the lowest and the highest values within 1.5 interquartile range (whiskers), and outliers (dots). Right side density plots show the distribution of data.

The parents of both sexes continued to feed the chicks after the switch and did so with a similar frequency as before the experiment (Table 3, Fig. 2). The chick switch did not affect the duration of the feeding events. Feeding events, either considered altogether (Table 3) or focusing on the first three only (Table 3, Fig. 3), were of a similar duration in both the experimental and control nests.

**Table 3:** Summary of the mixed generalized linear models [with Poisson (for 1) and gamma (for 2 and 3) error distribution] testing: (1) the number of feedings, (2) the duration of feeding events and (3) the duration of the first three feeding events performed during two 40-h recording sessions: before the experiment (control) and after the cross-fostering experiment (experimental). Sex and bird identity were included as fixed and random effects, respectively.

Predictors	Number of feedings				Duration of feeding events				Duration of first three feeding events			
	Estimates	SE	z	*p*	Estimates	SE	z	*p*	Estimates	SE	z	*p*
Intercept	1.74	0.12	14.58	< 0.0001	0.07	0.01	5.54	< 0.0001	0.05	0.01	4.21	< 0.0001
Session (control)	0.19	0.15	1.30	0.19	0.01	0.01	1.56	0.12	0.02	0.01	1.60	0.11
Sex (male)	− 0.12	0.16	− 0.78	0.43	− 0.01	0.02	− 0.55	0.59	− 0.01	0.01	− 0.45	0.65
Session x Sex	0.08	0.21	0.40	0.69	0.001	0.01	− 0.14	0.89	0.01	0.01	− 0.66	0.51

The experiment apparently did not affect the chick growth rate, as the peak and fledging body masses were similar in both the cross-fostered and control chicks (Table 4). Additionally, the duration of the nesting period, i.e., fledging age, was similar for the cross-fostered and control chicks (Table 4). Moreover, the physiological stress level expressed by the FCM concentrations was similar in the matched control and cross-fostered chicks (Table 5, Supplementary Fig. 1).

**Table 4:** Peak and fledging body masses and fledging age of the control and cross-fostered chicks.

Parameter	Cross-fostered chicks		Control chicks		Bootstrapped Welch Two Sample t-test	
	Mean ± SD	N	Mean ± SD	N	CI	*p*
Peak body mass (g)	125.57 ± 7.38	13	123.08 ± 9.97	16	− 8.49 to 3.63	0.44
Fledging body mass (g)	116.43 ± 16.63	11	111.85 ± 10.07	8	− 13.92 to 5.16	0.44
Fledging age of chicks (days)	26 ± 1.57	11	26 ± 0.83	8	− 0.84 to 1.21	0.70

**Table 5:** Summary of the linear model comparing the FCM levels (log-transformed values) in the control and cross-fostered chicks. Significant (p < 0.05) effects are bolded.

Predictors	Estimates	CI	*p*
Intercept	0.48	− 1.75 to 2.72	0.658
Group (control)	0.06	− 0.31 to 0.42	0.742
Chick age	0.15	0.04 to 0.27	**0.011**

Observations: 26; R^2^/R^2^ adjusted: 0.248/0.182.

## Discussion

All the little auk chick calls analysed were more similar when comparing their structure within each chick than between chicks. Additionally, the high individual repeatability of several acoustic parameters of chick calls demonstrates that little auk chick vocalizations encode some information about their individual identity. The Beecher information statistic values and subsequent calculations of the number of distinguished individuals further support these findings. Although the number of individuals who could potentially be differentiated based on the acoustic parameters (5–48) is not particularly high compared to other bird species (e.g., 433 in the colony-breeding cliff swallow, *Hirundo* *pyrrhonota*^5,27^), it is considerable and suggests that vocal cues may at least support vocal IR in the little auk.

Chick recognition in the little auk may be based on acoustic cues, although it does not exclude the possibility that other channels are also utilized in parent–offspring communication in the species (e.g., visual cues of the chick and nest). For instance, in territorial penguin species, the nest is used as a meeting point for parents and chicks, but vocal recognition systems are used in parallel. In Adélie penguin (*Pygoscelis* *adeliae*) and gentoo penguin (*Pygoscelis* *papua*), vocal recognition is based on the frequency spectrum, while in macaroni penguin (*Eudyptes* *chrysolophus*), it is based on temporal and frequency modulations^11,12^.

We found significant individual acoustic patterns, both in the early and late nesting periods of the little auk. However, the greatest individuality and repeatability measure we noted was for the older chicks (but not fledglings). The question of the ontogeny effect on vocalization should be further studied, but our results already demonstrate that the frequency ranges of calls increase with chick age, which could be a result of syrinx and trachea development^28^ since the fundamental frequency in birds usually correlates with the size of the syrinx^29^. Ontogeny may also be relevant to the higher food demands and increased mobility of the older chicks, since high frequency calls should encourage parents to feed the chicks and possibly decrease the aggression of adults towards chicks^30^. Overall, the occurrence of individuality cues in calls emitted by chicks should improve parents’ ability to find and recognize their chicks^17^. Thus, significant interindividual differences in chick calls should be regarded as an adaptation to IR.

It is known for other species that the individual features of chick vocalizations become more differentiated when the fledging time and/or maturity approaches^8,28,30,31^. Fledging (and postfledging) is a critical period of the little auks chick’s life^21^, and the father–chick vocal interactions are the most intensive at these times (*personal* *observation*). Thus, we expected to find evidence of high IR at this stage. However, although the content of information in the signal (as denoted by the Beecher statistic) and its repeatability increase with age and are of nonzero value at fledging, these individuality metrics are lower in fledging calls than in begging signals, which are produced earlier in the chick life. This is surprising but could be an effect of comparisons of different calls: begging vs. fledging. We selected types of calls that were the most typical for a given nesting stage, but it is possible that the functions of begging and fledging calls are different, and thus, IR is related to a given signal. Perhaps vocal recognition of the chick begging for food is more important, as the risk of misidentification is greater (especially at the later nesting stage) and the costs are also high (loss of food load). During fledging, the chick remains in close proximity to the parent, so its identity may be obvious. Then, the chick along with the parent flies to the sea not being accompanied by other birds, and the risk of chick misidentification is quite low. In the occasional event of parent and chick separation after a predator attack^21^, the IR based on the fledging call may still be sufficient, as this happens outside the colony area. The chick may also use different calls to reunite with the parent. Thus, a fledging call may primarily serve as a signal of the proximity of the fledgling (a high frequency of the call would support that function) and only secondarily for its identification. In addition, fledging signals may also provide information about chicks’ emotional state^32^ and readiness to fledge, which may mask acoustic identity but may be the most important information for the parent at this time.

Despite the detected strong individual characteristics in the chick calls, little auk parents accepted the cross-fostered chicks, continuing their care. The experimental chicks were not negatively affected because they had a similar growth rate and physiological stress level (evaluated by FCM concentrations) as the control chicks. Our results suggest that little auks do not discriminate between their own and cross-fostered offspring by vocal, visual, olfactory or behavioural cues. Likewise, in chicks of the burrow-nesting thin-billed prion *Pachyptila* *belcheri*, the acoustic parameters of begging calls were highly chick-specific^33,34^, even though parents also accepted cross-fostered chicks^33^. There are more examples of colonial birds accepting chicks that are not their own. For instance, in the little tern (*Sternula* *albifrons*), chicks sometimes switch the nest if the parents neglect their feeding duties^35^. The adoption of chicks has also been documented in the common tern (*Sterna* *hirundo*) and several gull species^36–38^. Most likely, the parents accepting a switched chick did not recognize that it had been switched. The IR mechanisms may not be important in avian species whose chicks remain in a burrow nest throughout the rearing period, since parents simply remember the location of the burrow (and a parent is usually absent when a chick departs its nest)^33^. Thus, in colonial species, some fostering behaviour may be a consequence of chick intermingling and failed IR.

However, it is also possible that parents recognize the chicks but accept foster parenthood. Adoption and alloparental care may have evolved as an adaptive feature for some avian species. When scarce breeding sites result in the formation of large breeding colonies, the chances for fostering an alien young arise, and thus, there is a higher possibility of chick recognition mistakes that may promote the occurrence of alloparental care and adoption^39^. In the common guillemot (*Uria* *aalge*), in which parent–chick recognition is well recognized from the very beginning of the nesting period, cases of adoption or alloparental care have been documented, not only in failed breeders but also in parents rearing two chicks simultaneously^40^. However, in thick-billed guillemots, cross-fostering experiments showed that the likelihood of adoption declined with increasing chick age^8^. In previous experiments with little auk chicks rotated among nests (started in early life), the growth and survival of the nestlings was not impaired by the experiment; surprisingly, the parent little auks were eager to care for two unrelated chicks in the nest^41^.

From an evolutionary standpoint, adults should avoid providing foster care, while chicks may benefit through such foster care, e.g., by obtaining more food^37^. Adult individuals, more closely related to their own offspring than to the offspring of their neighbours, should put their parental efforts into their own brood^4^. However, according to the theory of kin selection (Hamilton’s rule), foster parents may benefit by raising the offspring of a close relative^2,3^. For example, thick-billed guillemots that nest on close ledges showed a relatively high relatedness^42^. The occurrence of alloparental care and adoption in this species may be explained by the fact that the fitness benefits of foster care considerably exceeded the fitness costs^37^. Assuming that the variation in vocal signalling may be an outcome of genetic differences between individuals^34^, the genetic relatedness and voice similarity among neighbouring chicks could be tested in future studies.

Conversely, behavioural observations of adult little auks, i.e., noted acts of aggression towards foreign chicks wandering away from their natal nest (*personal* *observation*), question the possibility of intentional adoption in the species. Alloparental care is rather unusual in seabirds, and most species ignore each other’s offspring or act aggressively towards them. In thick-billed guillemots, conspecifics may attack unaccompanied chicks on the water, and this aggression is the most important cause of chick mortality at departure^43^. In territorial Adélie penguins, chicks of any age were attacked by other penguins as strongly as if they were intruding adults^9^. Since parents cannot rear more than two chicks, adult aggression towards wandering, foreign chicks may enhance the survival of their own chicks (in a few instances in which an adult Adélie penguin adopted a third chick, it resulted in death by starvation of its own smallest chick)^9^.

Nonetheless, the costs and benefits of foster care may vary between bird species and appear to be related to the duration of parental care^37^. Especially in long-living seabirds, occasional adoptions (i.e., acceptance errors) persist because the long-term reproductive cost of accepting a foreign chick outweighs the error-related costs of IR, which could result in the parent killing its own chicks^37^. Moreover, since adults encounter their own chick more frequently than foreign ones, selection will favour the universal acceptance of a chick found in the nest unless contextual evidence indicates that the chick is indeed foreign (e.g., the parent observed a foreign chick entering its nest)^37,44^. In the case of little auks, parents may recognize the nest site, and they may not suppose that the chick that they have found is not their own. Thus, most likely, any chick present in the nest and begging for food will be fed. The parent birds may disregard potential individual characteristics (calls, visual markings, odours) enabling the discrimination of offspring because of the high likelihood that normally the chick in the nest is indeed the native chick^44^.

Contrary to our predictions, little auk males were as equally likely to accept cross-fostered chicks as females, even though they would be expected to have a higher discrimination rate due to their postfledging interactions with chicks^20^. In contrast, male and female parents equally contribute to chick provisioning^22,45^ and spend a similar amount of time with the chick; thus, their ability to recognize the offspring may be similar. Interactions of male parents with offspring (including vocal IR) require further investigation to conclude the causality of the sex-specific behaviour of brood desertion (female) and chick accompaniment in its first flight into the sea (male). Additionally, parent interactions with offspring after colony departure are poorly known in the little auk and certainly deserve attention by researchers.

## Materials and methods

We conducted our study in a large breeding colony of little auks (77°00′N, 15°33′E), situated in the Hornsund fiord, in the southwestern part of Spitsbergen (Svalbard archipelago) in 2016 and 2017. The studied colony is one of the largest breeding aggregations of this species on Svalbard (400,000–590,000 pairs^46,47^), making the site representative of the little auk population. The cold water sea currents and relatively low sea surface temperature (SST) in the little auk foraging grounds in the study area are associated with a high abundance of energy-rich zooplankton species, such as copepods (e.g., *Calanus* *glacialis*), which are preferred by zooplanktivorous little auks^48–50^. This specific oceanographic location provides good breeding conditions for little auks, and thus, their vocalizing behaviour is unlikely to be heavily affected by environmental conditions, which could have masked the parent–offspring vocal interactions.

### Chick vocalization

Given the possibility of ontogenetic changes in chick vocalization, we aimed to record chick begging calls at the very beginning and at the end of the rearing period (capturing the signals that parents were exposed to during their presence in the nest with the chick; Fig. 1a,b), as well as chick fledging calls (potentially the key signal in parent–offspring recognition in the little auk; Fig. 1c). Thus, we recorded begging calls during continuous 48-h sessions at the 1st and 4th weeks of chick life and fledging calls from the 25th day of life until fledging. The audio recordings were associated with the video recording of the focal nests; thus, parent absence/presence could be controlled for the begging calls (i.e., only begging calls in the presence of a parent were considered). We used miniature microphones (38 × 14 mm) placed inside each focal nest (Olympus ME 51S, OM Digital Solutions GmbH, Hamburg, Germany) for the recording, connected to digital voice recorders (Olympus LS-3 and LS-P4, OM Digital Solutions GmbH, Hamburg, Germany) placed outside the nest. We performed all the recordings in WAV format (48 kHz/16 bit). A total of 538 good-quality begging calls were selected in the 1st week of life in 2017 (10 chicks × 28–79 calls per chick), 100 begging calls in the 4th week of life in 2017 (10 chicks × 10 calls per chick) and 446 fledging calls in 2016 (15 chicks × 15–66 calls per chick). We estimated the age of the chicks according to the hatching date, as nests were monitored daily, starting from late incubation until the chick had hatched.

For each selected call, we created spectrograms with a 512-sample Hamming window and 87.1% overlap, providing a time resolution of 1.50 ms and frequency resolution of 86.1 Hz using RAVEN Pro 1.6.1^51^. We visually classified the call types (begging/fledging call) based on their spectrographic structure. Since a single syllable of the little auk chick calls comprises one fundamental frequency with its associated harmonic series (Fig. 1a–c), we made the selections on the lowest frequency band, called the fundamental frequency. We imported the selection tables and the sound files into R software ver. 4.1.3^52^ using the Rraven package^53^. We described each call with several bioacoustic parameters measured on a single syllable of the begging or fledging call: duration of a syllable (Duration); top fundamental frequency (F max); bottom fundamental frequency (F min); peak fundamental frequency (F peak), representing frequency at the maximum amplitude; medium frequency (F medium), which divides the selection into two frequency intervals of equal energy; first (F Q_1_) and third (F Q_3_) quartiles of the frequency spectrum; bandwidth–interquartile frequency range (F IQR) and spectrographic entropy (Entropy) using the warbleR package^54^.

### Cross-fostering experiment

We conducted a cross-fostering experiment on 18 chicks in 2017 to test whether the parents are capable of accepting a chick that was not their own, i.e., to continue feeding after the chick switch. At the start of the experiment, the chicks were 11–14 days old, and we matched chicks in the pair to be exactly of the same age (i.e., 0-day difference) and with a similar body mass. We monitored the experimental nests by time-lapse video recordings (1 frame per second) during two consecutive 48-h sessions (later trimmed to 40 h to account for technical issues in some nests). The first session was performed just before the switch, and the second was performed just after. For reliable and efficient identification on the video material, as early as the late incubation stage, we individually marked both parents from the experimental nests with a colour combination of metal and plastic leg rings and colour signs painted on breast feathers. For this purpose, birds were captured by hand while in the nest during the late incubation period. The sex of focal parents was established molecularly^55^ based on a small blood or feather sample collected upon handling (either in a previous or the study season). We processed the video material with VLC software^56^ following established protocols^57,58^. Briefly, we considered the parent to be feeding if it entered the nest with food (indicated by a filled gull pouch) and exited it again without food. In total, we considered video material from 16 nests; we excluded two nests due to technical issues (recording errors).

To evaluate the experimental effect, we compared three chick growth parameters between experimental chicks (all that were available after switching, n = 13) and control chicks (n = 16). We established the following chick growth parameters based on regular weighing (every 3 days throughout the whole nesting period until chicks disappeared from the nest) with an electronic balance (0.01 g accuracy; Ohaus Europe GmbH, Nänikon, Switzerland):

- peak body mass (highest mass noted per chick),
- fledging body mass (last body mass measured before chick departure from the colony),
- age at fledging (day of the last presence in the nest).

All three parameters are considered effective growth indicators in the little auk^59^. The sample sizes differed for particular parameters since some chicks were not reachable on the day of measurements.

Additionally, to establish the impact of the cross-fostering experiment on the chicks’ body condition, we examined the physiological stress level (based on faecal corticosterone metabolites, FCMs^60^) in the experimental and control chicks. We collected chick faecal samples during weighing and immediately placed them into plastic tubes that were labelled and kept in a field cooler box with frozen gel packs for up to 2 h. The samples were then stored in a freezer at − 20 °C until analysis. We sampled each chick only once, considering its age on the day of sampling (since FCM levels increase with chick age^49^). We measured the FCMs with an 11-oxoaetiocholanolone enzyme immunoassay^61^ as described in earlier studies on little auks^49,62^.

All animal research protocols were carried out in accordance with guidelines for the use of animals^63^ and approved by the Norwegian Animal Research Authority and the Governor of Svalbard.

### Statistical analysis

To check whether the call structure of chicks could act as a key for parents to recognize their own chicks, we compared the structure of chick begging and fledging calls within and between individual chicks with three approaches: (a) testing the repeatability of various bioacoustic parameters, (b) comparing the call structure using spectrographic cross-correlations^64^ and (c) calculating the Beecher information statistic (H_s_)^65,66^. For the repeatability analysis, we considered each call type and each of the bioacoustic parameters separately in search of the one that would be the key for vocal recognition; the parameter with the highest repeatability would be the one most important for the bioacoustic identity. We tested the repeatability of the parameters with generalized linear mixed-effects models (formula: parameter ~ chick identity) fitted by the restricted maximum likelihood (REML) using the rptR package^67^. We set the number of parametric bootstraps for an interval estimation to 1000 and the number of permutations used for calculating asymptotic *p* values to 1000. We denoted *p* values from significance tests based on likelihood ratios.

To compare call structures with spectrographic cross-correlations (Pearson correlation), we calculated a mean pairwise cross-correlation for each individual using the warbleR package^54^. Then, to assess the similarities of calls, we created pairwise binary matrices when assessing individual signatures (with 0 to denote the same individual and 1 to denote a different individual). We used the r statistic of the Mantel test (100,000 iterations), calculated in the vegan package^68^, as a similarity measure^69^. Statistical significance indicates that the parameters are more similar within individuals than between individuals.

To measure the level of individuality coded within chick vocalizations, we applied the information theory approach proposed by Beecher^65^. The Beecher information statistic (H_s_) has been recently recommended as a gold standard individual identity metric because it is easily calculated, has superior performance with respect to other metrics, and can be used to quantify identity information in a complex signal, indicating the number of individuals that can be discriminated given a set of measurements^66^. To calculate H_s_ for each chick life stage separately (as the sets are partially independent), we used the calcHS() function from the IDmeasurer package^66^, applied to nine principal components derived from the data (principal component analysis performed using the calcPCA() function from the IDmeasurer package^66^). Using H_s_ and the formula: $2^{{{\text{H}}_{{\text{s}}} }}$, we also calculated a maximum number of individuals that could be unambiguously acoustically discriminated based on the data^65,66^.

To analyse the behaviour of the parents after chick switching, we compared three behavioural parameters between the control and experimental sessions. First, we modelled the number of feedings performed by a parent during 40 h (response variable) with the session (control/experiment), sex of the parent and interaction between the two (fixed factors) and bird identity as a random factor using the generalized linear mixed model (GLMM) with Poisson distribution. We included sex (and interaction in the model) due to a possible sex effect on parent behaviour. Second, we investigated the duration of feeding events, expecting that parents facing foreign chicks may need more time to recognize/accept/feed them. To this end, we first established the duration of time intervals between the parent’s arrival with food to the nest (its first appearance, after a longer period of absence; with food in the gular pouch) and its very first exit from the nest without food. Thus, feeding events were complex and included latency to approach the nest, multiple entries/exits into the nest, and time spent in the nest. Among these elements, the latter two are likely to be relevant to the question of parent–offspring recognition, while latency should be the same for both control and experimental circumstances (it would be difficult to exclude latency, however). Thus, the duration of the feeding events (response) was modelled with session (control/experiment), sex of parent and their interaction (fixed factors) and bird identity as a random factor using GLMM with gamma error distribution. Finally, to investigate the duration of feeding events at a finer scale, considering the duration of only the first three feeding events and expecting that later the parents may simply accept the chick and treat it normally, while at the first visits the parent’s behaviour would be different, we used the same GLMM construct as for the full dataset (duration of all feeding events).

We compared chick growth parameters (peak and fledging body mass, as well as chick fledging age) between the experimental and control groups with the bootstrapped Welch two-sample t test using the MKinfer package^70^. We tested differences in log-transformed FCM levels between control and cross-fostered chicks using a linear model with group (control/experiment) and chick age as fixed factors. We performed mixed models using the lme4 package. Before analyses, we checked whether the data sufficiently met relevant assumptions using Q–Q plots (quantile expected in normal distribution vs. quantile observed plot for residuals).

We performed all statistical analyses in R software version 4.1.3^52^.

### Ethics approval and consent to participate

All applicable international rules for the use of animals, as specified in the guideline of the Association for the Study of Animal Behaviour, were followed. Besides birds were captured and handled under permissions issued by the Norwegian Animal Research Authority (7/66141) and the Governor of Svalbard (16/00770-3, 17/00663-2).

### Supplementary Information

Supplementary Table 1.

Supplementary Figure 1.

## Data Availability

The datasets are available from the corresponding author on request. Call samples are available at https://osf.io/dfbxu/?view_only=bf707a44e76e4aac9ea81b3e4a33b515.

## Abbreviations

IR: Individual recognition
FCM: Faecal corticosterone metabolites

## References

[1] Gardner A, Foster KR, Korb J, Heinze J (2008). The evolution and ecology of cooperation—History and concepts. Ecology of Social Evolution.

[2] Hamilton WD (1964). The genetical evolution of social behaviour. II. J. Theor. Biol..

[3] Hamilton WD (1964). The genetical evolution of social behavior. I. J. Theor. Biol..

[4] Evans RM, Burger J, Olla BL, Winn HE (1980). Development of behavior in seabirds: An ecological perspective. Behavior of Marine Animals.

[5] Medvin MB, Stoddard PK, Beecher MD (1993). Signals for parent-offspring recognition: A comparative analysis of the begging calls of cliff swallows and barn swallows. Anim. Behav..

[6] Riou S, Chastel O, Hamer KC (2012). Parent–offspring conflict during the transition to independence in a pelagic seabird. Behav. Ecol..

[7] Kidawa D (2015). Parental efforts of an Arctic seabird, the little auk *Alle*
*alle*, under variable foraging conditions. Mar. Biol. Res..

[8] Lefevre K, Montgomerie R, Gaston AJ (1998). Parent–offspring recognition in thick-billed murres (Aves: Alcidae). Anim. Behav..

[9] Spurr EB (1975). Behavior of the Adélie penguin chick. Condor.

[10] Lengagne T, Lauga J, Aubin T (2001). Intra-syllabic acoustic signatures used by the king penguin in parent-chick recognition: An experimental approach. J. Exp. Biol..

[11] Aubin T, Jouventin P (2002). How to vocally identify kin in a crowd: The penguin model. Adv. Study Behav..

[12] Jouventin P, Aubin T (2002). Acoustic systems are adapted to breeding ecologies: Individual recognition in nesting penguins. Anim. Behav..

[13] Jones IL, Falls JB, Gaston AJ (1987). Vocal recognition between parents and young of ancient murrelets, *Synthliboramphus*
*antiquus* (Aves: Alcidae). Anim. Behav..

[14] Beecher MD, Beecher IM, Hahn S (1981). Parent–offspring recognition in bank swallows (*Riparia*
*riparia*): II. Development and acoustic basis. Anim. Behav..

[15] Tibbetts EA, Dale J (2007). Individual recognition: It is good to be different. Trends Ecol. Evol..

[16] Bonadonna F, Villafane M, Bajzak C, Jouventin P (2004). Recognition of burrow’s olfactory signature in blue petrels, *Halobaena*
*caerulea*: An efficient discrimination mechanism in the dark. Anim. Behav..

[17] Falls BJ, Kroodsma DE, Miller EH (1982). Individual recognition by sound in birds. Acoustic Communication in Birds, Vol. 2: Song Learning and its Consequences.

[18] Mathevon N, Charrier I, Jouventin P (2003). Potential for individual recognition in acoustic signals: A comparative study of two gulls with different nesting patterns. Comptes Rendus Biol..

[19] Gaston AJ, Jones IL (1998). The Auks: Alcidae. Bird Families of the World.

[20] Insley SJ, Paredes R, Jones IL (2003). Sex differences in razorbill *Alca*
*torda* parent-offspring vocal recognition. J. Exp. Biol..

[21] Stempniewicz L, Vani M (2001). *Alle**alle* little auk. The Journal of the Birds of the Western Palearctic, BWP Update.

[22] Wojczulanis-Jakubas K, Jakubas D (2012). When and why does my mother leave me? The question of brood desertion in the dovekie (*Alle*
*alle*). Auk.

[23] Wojczulanis-Jakubas K (2020). Duration of female parental care and their survival in the little auk *Alle*
*alle*—Are these two traits linked?. Behav. Ecol. Sociobiol..

[24] Wojczulanis-Jakubas K, Jakubas D, Stempniewicz L (2022). The little auk *Alle*
*alle*: An ecological indicator of a changing Arctic and a model organism. Polar Biol..

[25] Wojczulanis-Jakubas K, Jakubas D, Øigarden T, Lifjeld JT (2009). Extrapair copulations are frequent but unsuccessful in a highly colonial seabird, the little auk, *Alle*
*alle*. Anim. Behav..

[26] Stempniewicz L (1981). Breeding biology of the Little Auk, *Plautus*
*alle* in the Hornsund region, SW Spitsbergen. Acta Ornithol..

[27] Beecher M, Medvin M, Stoddard P (1986). Acoustic adaptations for parent–offspring recognition in swallows. Exp. Biol..

[28] Goncharova MV, Klenova AV, Bragina EV (2015). Development of cues to individuality and sex in calls of three crane species: When is it good to be recognizable?. J. Ethol..

[29] Suthers RA, Zollinger SA (2004). Producing song: The vocal apparatus. Ann. N. Y. Acad. Sci..

[30] Klenova AV (2010). Voice breaking in adolescent red-crowned cranes (*Grus*
*japonensis*). Behaviour.

[31] Klenova AV, Goncharova MV, Bragina EV, Kashentseva TA (2014). Vocal development and voice breaking in demoiselle cranes (*Anthropoides*
*virgo*). Bioacoustics.

[32] Osiecka AN, Briefer EF, Kidawa D, Wojczulanis-Jakubas K (2023). Seabird’s cry: Repertoire and vocal expression of contextual valence in the little auk (*Alle*
*alle*). Sci. Rep..

[33] Quillfeldt P, Masello JF, Strange IJ, Buchanan KL (2006). Begging and provisioning of thin-billed prions, *Pachyptila*
*belcheri*, are related to testosterone and corticosterone. Anim. Behav..

[34] Duckworth A, Masello JF, Mundry R, Quillfeldt P (2009). Functional characterization of begging calls in thin-billed prions *Pachyptila*
*belcheri* chicks. Acta Ornithol..

[35] Saino N, Fasola M, Crucicchia E (1994). Adoption behavior in little and common terns (Aves; Sternidae): Chick benefit and parent ’ fitness costs. Ethology.

[36] Holley AJF (1984). Adoption, parent-chick recognition and maladaptation in the herring gull *Larus*
*argentatus*. Z. Tierpsychol..

[37] Brown KM (1998). Proximate and ultimate causes of adoption in ring-billed gulls. Anim. Behav..

[38] Morris RD, Woulfe M, Wichert GD (1991). Hatching asynchrony, chick care, and adoption in the common tern: Can disadvantaged chicks win?. Can. J. Zool..

[39] Riedman MLML (1982). The evolution of alloparental care and adoption in mammals and birds. Q. Rev. Biol..

[40] Wilson LJ, Birkhead TR (2001). Adoption of the common guillemot *Uria*
*aalge*. Atl. Seabirds.

[41] Gębczyński A, Taylor JRE, Konarzewski M (1996). Growth of dovekie (*Alle*
*alle*) chicks under conditions of increased food demand at the nest: Two field experiments. Can. J. Zool..

[42] Friesen VL, Montevecchi WA, Baker AJ, Barrett RT, Davidson WS (1996). Population differentiation and evolution in the common guillemot *Uria*
*aalge*. Mol. Ecol..

[43] Gilchrist HG, Gaston AJ (1997). Factors affecting the success of colony departure by thick-billed murre chicks. Condor.

[44] Beecher MD (1991). Successes and failures of parent-offspring recognition in animals. Kin Recognition.

[45] Wojczulanis-Jakubas K, Jakubas D, Kidawa D, Kośmicka A (2012). Is the transition from biparental to male-only care in a monogamous seabird related to changes in body mass and stress level?. J. Ornithol..

[46] Isaksen K, Isaksen K, Bakken V (1995). The breeding population of Little Auk (*Alle**alle*) in colonies in Hornsund and northwestern Spitsbergen. Seabird Populations in the Northern Barents Sea.

[47] Keslinka LK, Wojczulanis-jakubas K, Jakubas D, Neubauer G (2019). Determinants of the little auk (*Alle*
*alle*) breeding colony location and size in W and NW coast of Spitsbergen. PLoS Biol..

[48] Kwasniewski S (2010). The impact of different hydrographic conditions and zooplankton communities on provisioning Little Auks along the West coast of Spitsbergen. Prog. Oceanogr..

[49] Kidawa D (2014). Variation in faecal corticosterone metabolites in an Arctic seabird, the Little Auk (*Alle*
*alle*) during the nesting period. Polar Biol..

[50] Balazy K, Trudnowska E, Błachowiak-Samołyk K (2019). Dynamics of *Calanus* copepodite structure during little auks’ breeding seasons in two different Svalbard locations. Water.

[51] Center for Conservation Bioacoustics. Raven Pro: Interactive sound analysis software (Version 1.6.1) [Computer software]. https://ravensoundsoftware.com (The Cornell Lab of Ornithology, 2019).

[52] R Core Team. R: A language and environment for statistical computing. http://www.R-project.org/ (R Foundation for Statistical Computing, 2021).

[53] Araya-Salas, M. Rraven: Connecting R and Raven Sound Analysis Software (version 1.0.8). https://CRAN.R-project.org/package=Rraven (2017).

[54] Araya-Salas M, Smith-Vidaurre G (2017). warbleR: An R package to streamline analysis of animal acoustic signals. Methods Ecol. Evol..

[55] Jakubas D, Wojczulanis K (2007). Predicting the sex of Dovekies by discriminant analysis. Waterbirds.

[56] VideoLan. VideoLan.pdf. VLC media player. Retrieved from https://www.video (2006).

[57] Grissot A (2019). Parental coordination of chick provisioning in a planktivorous arctic seabird under divergent conditions on foraging grounds. Front. Ecol. Evol..

[58] Wojczulanis-Jakubas K (2022). Post-foraging in-colony behaviour of a central-place foraging seabird. Sci. Rep..

[59] Jakubas D (2013). Foraging closer to the colony leads to faster growth in little auks. Mar. Ecol. Prog. Ser..

[60] Palme R (2019). Non-invasive measurement of glucocorticoids: Advances and problems. Physiol. Behav..

[61] Möstl E, Maggs JL, Schrötter G, Besenfelder U, Palme R (2002). Measurement of cortisol metabolites in faeces of ruminants. Vet. Res. Commun..

[62] Kidawa D, Barcikowski M, Palme R (2017). Parent-offspring interactions in a long-lived seabird, the Little Auk (*Alle*
*alle*): Begging and provisioning under simulated stress. J. Ornithol..

[63] Guidelines for the Use of Animals. Guidelines for the treatment of animals in behavioural research and teaching. *Anim.**Behav.* **83**, 301–309 (2012).

[64] Khanna H, Gaunt SLL, McCallum DA (1997). Digital spectrographic cross-correlation: Tests of sensitivity. Bioacoustics.

[65] Beecher MD (1989). Signalling systems for individual recognition: An information theory approach. Anim. Behav..

[66] Linhart P (2019). Measuring individual identity information in animal signals: Overview and performance of available identity metrics. Methods Ecol. Evol..

[67] Stoffel MA, Nakagawa S, Schielzeth H (2017). rptR: Repeatability estimation and variance decomposition by generalized linear mixed-effects models. Methods Ecol. Evol..

[68] Dixon P (2003). VEGAN, a package of R functions for community ecology. J. Veg. Sci..

[69] Araya-Salas M (2019). Social group signatures in hummingbird displays provide evidence of co-occurrence of vocal and visual learning. Proc. R. Soc. B.

[70] Kohl, M. _MKinfer: Inferential Statistics_. R package version 1.1. https://www.stamats.de (2023).

