# Supplementary material for: Mine or my neighbours’ offspring: an experimental study on parental discrimination of offspring in a colonial seabird, the little auk Alle alle
Source: Sci Rep. 2023 Sep 12;13:15088. doi: 10.1038/s41598-023-41925-5 (PMC10497497; doi:10.1038/s41598-023-41925-5)
Supplement: Supplementary file 1 — Supplementary Table 1. [file 41598_2023_41925_MOESM1_ESM.pdf]

Supplementary Table 1. Summary of the linear models comparing different acoustic parameters of little auk chick begging calls at two stages: in the 1st and 4th weeks of life (the chick identity was included as a random effect; N observations = 638).

	Intercept (Begging calls in 1st week)			Begging calls in 4th week			Marginal R^2/ Conditional R^2	ICC of a random effect (chick)
Parameter	Estimates	CI	*p*	Estimates	CI	*p*		
Duration [s]	0.30	0.22 – 0.38	**<0.001**	-0.15	-0.17 – -0.13	**<0.001**	0.090 / 0.816	0.80
F min [Hz]	0.74	0.67 – 0.82	**<0.001**	0.43	0.41 – 0.46	**<0.001**	0.483 / 0.807	0.63
F Q_1 [Hz]	1.49	1.33 – 1.64	**<0.001**	0.81	0.75 – 0.86	**<0.001**	0.405 / 0.760	0.60
F medium [Hz]	1.64	1.46 – 1.83	**<0.001**	0.97	0.91 – 1.04	**<0.001**	0.416 / 0.783	0.63
F Q_3 [Hz]	1.73	1.52 – 1.95	**<0.001**	1.18	1.11– 1.25	**<0.001**	0.459 / 0.834	0.69
F max [Hz]	2.01	1.75 – 2.26	**<0.001**	1.37	1.30 – 1.44	**<0.001**	0.472 / 0.868	0.75
F IQR [Hz]	0.25	0.16 – 0.34	**<0.001**	0.37	0.34 – 0.40	**<0.001**	0.311 / 0.751	0.64
F peak [Hz]	1.60	1.39 – 1.81	**<0.001**	1.14	1.04– 1.24	**<0.001**	0.373 / 0.684	0.50
Entropy	0.79	0.76 – 0.81	**<0.001**	0.04	0.03– 0.05	**<0.001**	0.061 / 0.657	0.64
