# Supplementary material for: Mine or my neighbours’ offspring: an experimental study on parental discrimination of offspring in a colonial seabird, the little auk Alle alle
Source: Sci Rep. 2023 Sep 12;13:15088. doi: 10.1038/s41598-023-41925-5 (PMC10497497; doi:10.1038/s41598-023-41925-5)
Supplement: Supplementary file 2 — Supplementary Figure 1. [file 41598_2023_41925_MOESM2_ESM.pdf]

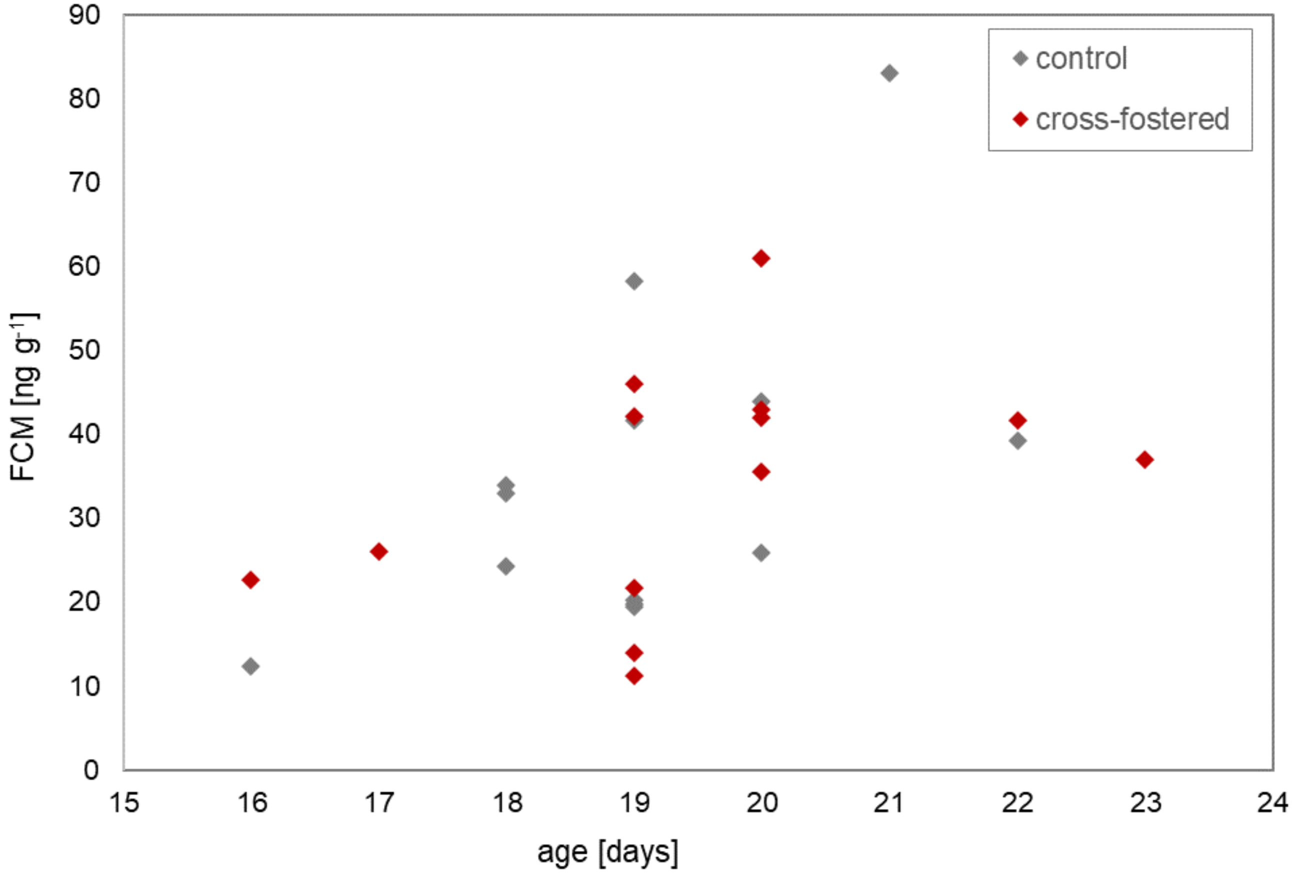

Supplementary Figure 1. Relationship between little auk chick FCM level and age in the control and cross-fostered group (N = 26, i.e. 13 control and 13 cross-fostered chicks).
